# Supplementary material for: Impact of COVID-19 lockdown on air quality analyzed through machine learning techniques
Source: PeerJ Comput Sci. 2023 Mar 31;9:e1270. doi: 10.7717/peerj-cs.1270 (PMC10280446; doi:10.7717/peerj-cs.1270)
Supplement: File S2 [file peerj-cs-09-1270-s002.docx]

**Outliers can be detected using visualization, implementing mathematical formulas on the dataset, or using the statistical approach, we used Scatter plot and Mahalanobis Distance using python**

**Scatter plot for PM2.5 pollutant**


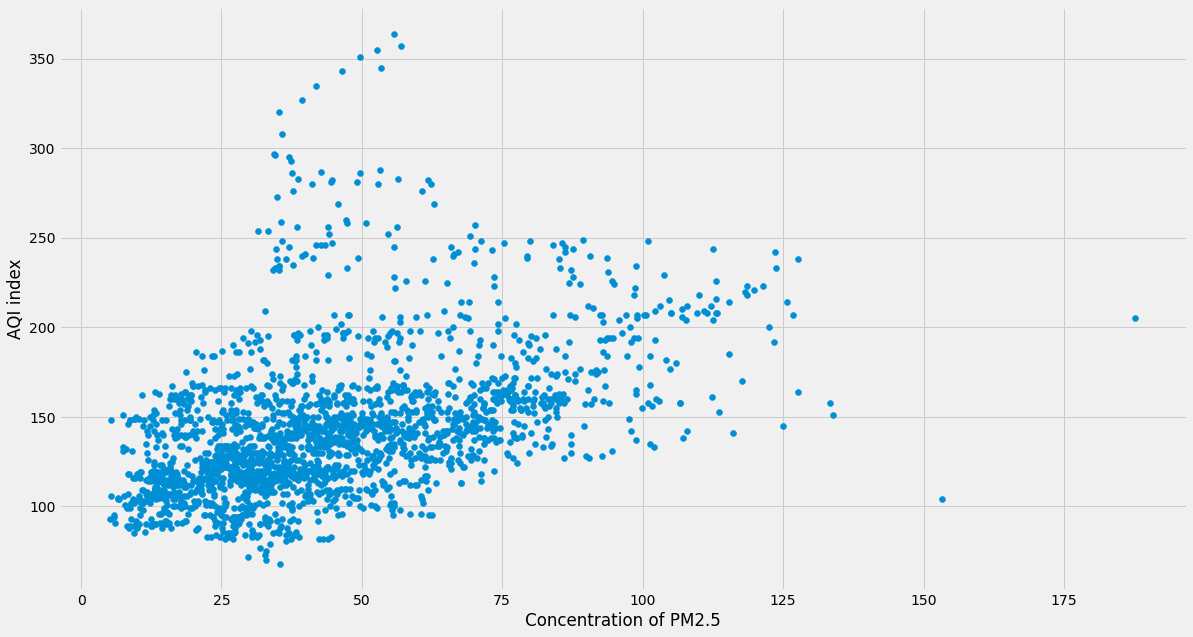


Figure 1: scatter plot for pm2.5

**Scatter plot for PM10**


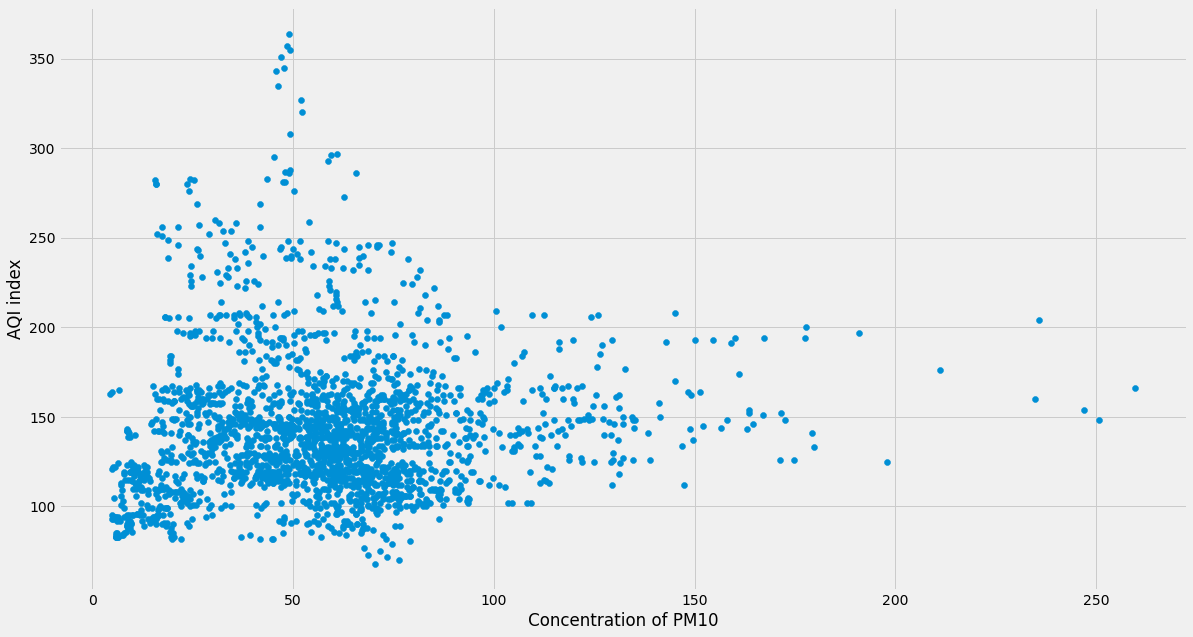


Figure 2: scatter plot for PM10

**Scatter plot for NO2 pollutant**


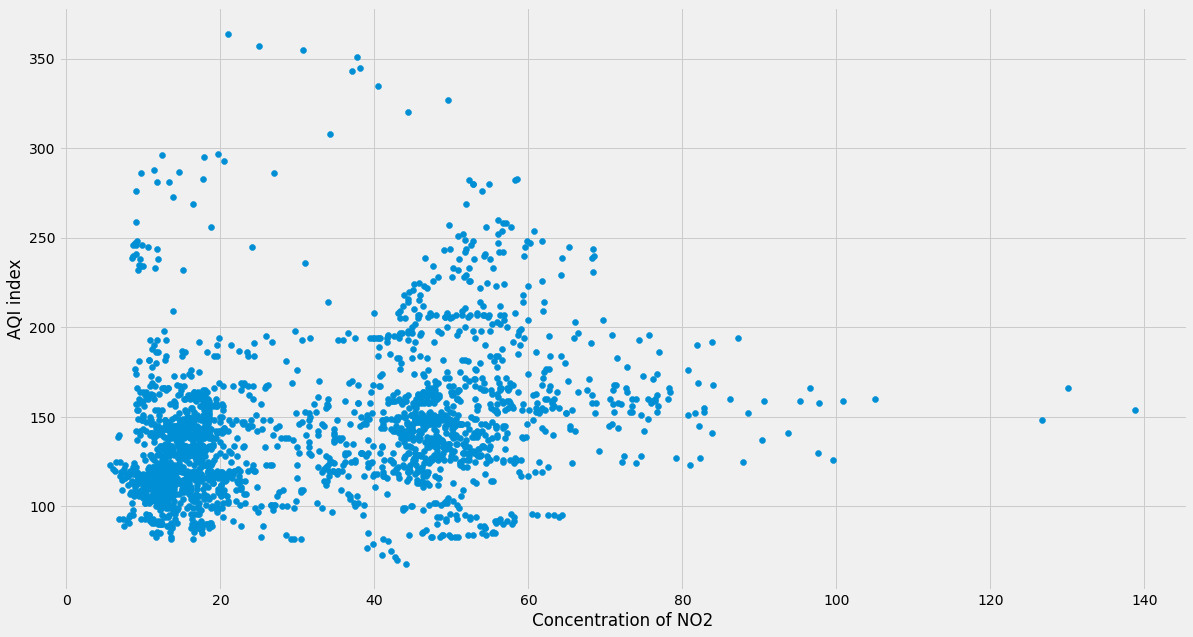


Figure 3: scatter plot for NO2

**Scatter plot for O3 pollutant**


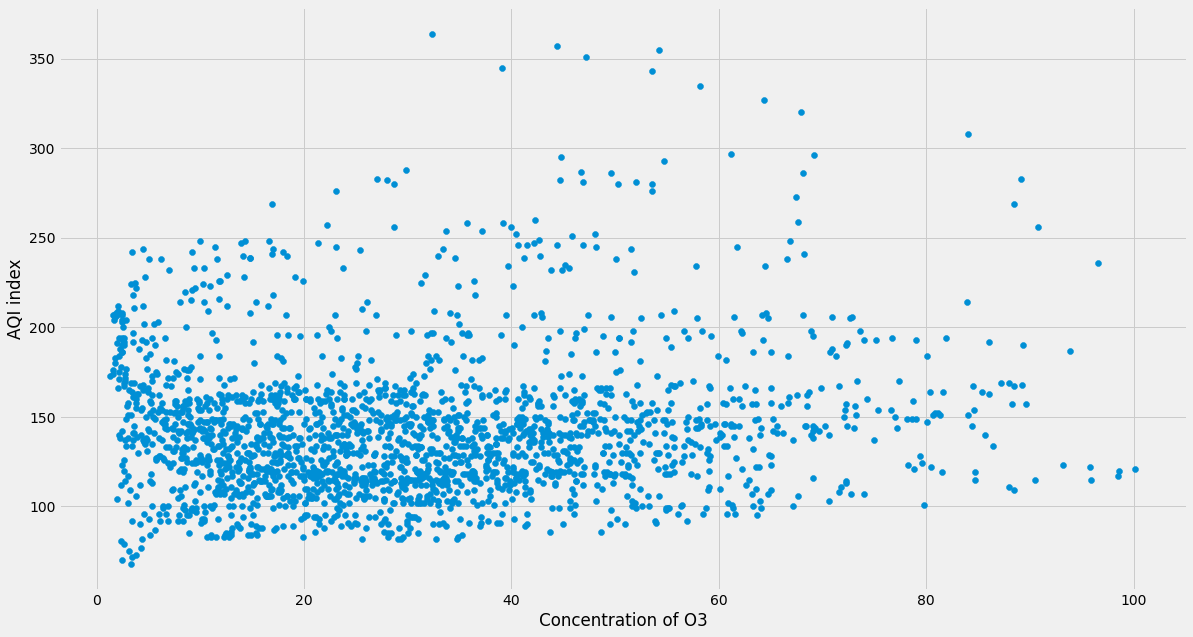


Figure 4: scatter plot for O3

**Mahalanobis Distance for PM2.5**


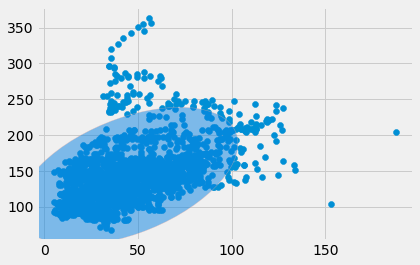


Figure 5: Mahalanobis Distance for PM2.5

Figure 5 shows the Mahalanobis Distance for PM2.5, the points stay outside the ellipse detected as outliers, this ellipse represents the area that wraps non-outlier values according to Mahalanobis Distance.

**Mahalanobis Distance for PM10**


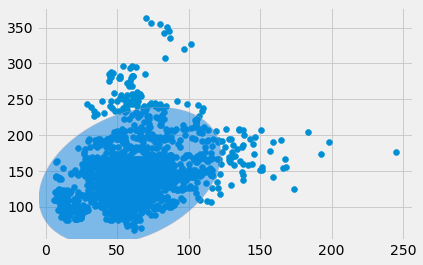


Figure 6: Mahalanobis Distance for PM10

Figure 6 shows the Mahalanobis Distance for PM10, the points stay outside the ellipse detected as outliers, this ellipse represents the area that wraps non-outlier values according to Mahalanobis Distance.

**Mahalanobis Distance for NO2**


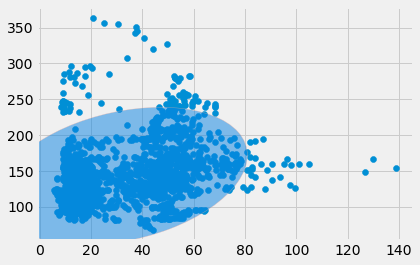


Figure 7: Mahalanobis Distance for NO2

Figure 7 shows the Mahalanobis Distance for NO2, the points stay outside the ellipse detected as outliers, this ellipse represents the area that wraps non-outlier values according to Mahalanobis Distance.

**Mahalanobis Distance for O3**


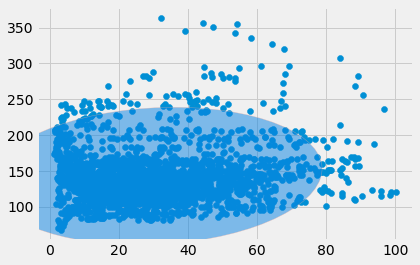


Figure 8: Mahalanobis Distance for O3

Figure 8 shows the Mahalanobis Distance for O3, the points stay outside the ellipse detected as outliers, this ellipse represents the area that wraps non-outlier values according to Mahalanobis Distance.
